# Intraocular reflectance of the ocular fundus and its impact on increased retinal hazard

**DOI:** 10.1016/j.zemedi.2022.03.001

**Published:** 2022-05-23

**Authors:** Nicole Fehler, Christian Lingenfelder, Sebastian Kupferschmid, Martin Hessling

**Affiliations:** a Institute of Medical Engineering and Mechatronics, Ulm University of Applied Sciences, Albert-Einstein-Allee 55, 89081, Ulm, Germany; b Pharmpur GmbH, Messerschmittring 33, 86343, Koenigsbrunn, Germany; c Clinic of Ophthalmology, Bundeswehrkrankenhaus Ulm, Oberer Eselsberg 40, 89081, Ulm, Germany

**Keywords:** Retinal hazard, Reflectance, Pigmentation, Intraocular illumination, Photochemical hazard, Thermal hazard

## Abstract

**Purpose:**

Inside the eye light can be reflected multiple times due to light-tissue interactions and the spherical geometry of the eye. Due to these optical properties, a defined retinal area is not only illuminated by direct light but also by indirect, reflected light from the inner side of the eyewall. During illumination for ophthalmic surgery, this could lead to an unintended increase in intraocular retinal irradiance, which was already discussed in previous studies but without a detailed consideration of spectral differences and a potential influence of pigmentation. In this study this effect is investigated wavelength-dependent to see if different wavelengths lead to different increase in irradiance, with a special focus on the raise in photochemical and thermal hazard to the retina. It is also examined whether this effect is dependent on the pigmentation of the eye.

**Methods:**

The reflectance properties of either less or highly pigmented porcine eyes are measured in the wavelength range between 350 and 1100 nm with an integrating sphere and a spectrometer. With these reflectance spectra the wavelength-dependent Sphere Multiplier *M* of porcine eyes can be calculated, which represents the increase of radiance due to multiple reflections inside a sphere compared to a planar diffuser of the same size. Based on measurements of the emitted irradiance of ophthalmic illumination fibers the increase in photochemical and thermal retinal hazard due to these multiple reflections is calculated for eyes with small and high amounts of pigmentation.

**Results:**

The reflectance of the inner eyewall in the range between 350 and 1100 nm is significantly higher for eyes with low pigmentation (between 4.90% and 37.44% reflectance) in comparison to eyes with a high amount of pigmentation (between 4.30% and 28.88% reflectance). The Sphere Multiplier for the inner side of the eyewall (sclera, choroid and retina) ranges between 1.13 and 1.59 and between 1.13 and 1.48 for eyes with low and high pigmentation, respectively, in the range between 350 and 1100 nm. The reflectance, as well as the Sphere Multiplier, is strongly wavelength-dependent due to the absorption spectra of melanin and hemoglobin, which are located in the eye. With increasing wavelength, the reflection properties and the Sphere Multiplier also increases. With this, the photochemical retinal hazard of highly pigmented eyes increases by (14.11 ± 0.09)% and of lightly pigmented eyes by (16.75 ± 0.35)% compared to if the reflection properties are not considered. The thermal retinal hazard increases by (14.30 ± 0.07)% for highly pigmented eyes and by (19.65 ± 0.17)% for low pigmented eyes.

**Conclusion:**

This study demonstrates that the anatomy and pigmentation of the eye plays an important role for the reflectance properties of the eye and for the photochemical and thermal hazard to the retina.

## Introduction

For pars plana vitrectomy and for visualisation of structures inside the eye a bright illumination of the ocular fundus is essential. Therefore, an endoillumination fiber is inserted into the eye and illuminates it from the inside [1]. The surgeon can guide this fiber and therefore change the field of view inside the eye and also the distance from the fiber tip to the retina. Typical distances between the fiber tip and the retina are 5 to 7 mm [2], but smaller distances are also possible and sometimes necessary. The smaller the distance from the fiber tip to the retina the more hazardous the illumination becomes to the retina [2], [3], [4]. By inserting the fiber into the eye, the retina is exposed to the complete spectrum, which is emitted by the fiber. With this method, no tissue like cornea, lens and aqueous humor, which otherwise act as natural filters for the harmful short-wavelength light [5], protects the retina from possible damage. If the retina is exposed to visible light photochemical and thermal damages can occur. In the standard DIN EN ISO 15004-2:2007 irradiation limits for preventing potential photochemical and thermal hazard to the retina, E_A-R_ and E_VIR-R_, are given [6]. If the limit value of E_A-R_ is exceeded, the maximal exposition time has to be considered. This time indicates the point at which a potential photochemical hazard to the retina is reached due to irradiation. The calculation of this period is also given in [6]. In this standard, these limit values are measured outside the eye on a surface, with which light can interact only once. However, the geometric anatomy of the eye is not considered. Due to its spherical shape, light can be reflected and scattered several times inside the eye before it is completely absorbed by the eyewall. With this, a defined spot on the eyewall is illuminated directly and additionally indirectly due to multiple diffuse reflections inside the eye due to the reflection properties of the different layers of the eyewall. These multiple reflections would lead to an increase of irradiance on a defined spot and the limit values given in [6] are reached faster.

The eyewall consists of three main layers, the sclera (outermost layer), the choroid (median layer) and the retina (innermost layer). These layers contribute to the reflectance properties inside the eye. Preece and Claridge (2002) defined the ILM (inner limiting membrane), RPE (retinal pigment epithelium), choroid and sclera as reflective structures within the eyewall [7]. However, since the ILM accounts for less than 1% of the reflection, it can be neglected [8]. The neural retina does not reflect light as its structures acts as optical light guide [9]. Previous measurements of fundus reflection have already been carried out in animal and human eyes and determined an increase in fundus reflection for increasing wavelength [7], [10], [11], [12]. In most examinations, reflectance measurements were taken from outside the eye through the anterior parts of the eye. Under these circumstances, data may be distorted due to reflections from the cornea and lens and due to their transmission properties. The latter were probably not included in the evaluation, since nothing is written about them in these publications. To avoid this problem Sayler et al. (2005) illuminated ex-vivo porcine eyes from the inside of the eye by inserting an illumination fiber in the vitreous of the eye [13]. With this set-up, an increase in reflectance of the inner eyewall with increasing wavelength was presented, too. However, only a relative reflectance spectrum was determined and no absolute reflectance values were given.

The eyewall contains a high amount of pigments, such as melanin and haemoglobin [14]. These pigments are found in the choroid and in the RPE of the eye and their absorption properties can influence the reflection spectra of the inner eyewall. Melanin also exists in the sclera, but only in very small amounts [15]. The color of the iris is related to the amount of melanin in the choroid [16], [17]. Choroidal melanocytes from eyes with dark colored irises exhibit a significant higher amount of melanin than from eyes with light colored irises. Menon et al. (1992) demonstrated a higher amount of melanin in the choroid-RPE for brown compared to blue ex-vivo human eyes [15], [18]. Previous measurements of the influence of pigmentation on the reflectance spectra reveal that a higher reflectance is observed for less pigmented eyes compared to more pigmented eyes [11], [19]. For the measurement of Delori and Pflibsen (1989) [19] the reflectance of only five eyes from either less or more pigmented people are examined and for the measurements of Flowers et al. (1977) [11], however, only one subject per pigmentation was examined, which is not sufficient to provide meaningful results.

Another influence on the reflection properties of the eye is caused by hemoglobin. Hemoglobin is mainly located in the choroid of the eye to provide nourishment to the retina and for thermal regulation of the eye [20], [21]. Literature spectra of porcine hemoglobin are very sparse. The extinction coefficient of human and porcine hemoglobin was investigated by Grosenbaugh et al. (1997) but only in a range of 600 to 1000 nm [22]. An investigation of purified porcine blood in the range of 450 and 700 nm was performed by Zhang et al. (2012) [23].

Due to the reflection properties of the ocular fundus and its spherical shape the intraocular irradiance may increase compared to if the eye would be unfolded. With the spherical geometry of the eye light can often hit the same part of the retina and therefore increase its hazard. Koelbl et al. (2019) supported this hypothesis and demonstrated that the spherical geometry of the porcine eye increases the irradiance on the surface of up to 1.5 compared to a reference measurement, where no multiple reflections are possible [24]. This value was not spectrally resolved but is a general value for the increasing radiation with a specific illumination set-up. A similar behavior is explained by the Sphere Multiplier *M* of an integrating sphere given in [25]. This parameter represents the increase of irradiance of an integrating sphere compared to a planar diffuser of the same size, due to multiple internal reflections inside the sphere. The Sphere Multiplier depends on the reflection properties of the sphere. Because the reflection properties depend on the wavelength, *M* is also wavelength-dependent. It is necessary to know the wavelength-dependence of the Sphere Multiplier as it plays an important role in increasing photochemical and thermal retinal hazard. Photochemical hazard to the retina is caused by short-wavelength blue and ultraviolet light [26], [27], [28]. On the other hand, thermal damage occurs in the visible to infrared wavelength region. Therefore, it is important to differentiate the Sphere Multiplier for these two wavelength ranges. With this, the increase in photochemical and thermal retinal hazard due to the reflection properties of the eye can be calculated.

To determine the wavelength-dependence of the effect of increasing intraocular irradiance in this study, the reflection properties of the inner eyewall of ex-vivo porcine eyes are investigated directly with an integrating sphere, without prior absorption, reflection or scattering of the light beam by other ocular tissue. The measurements are performed in the wavelength range between 350 – 1100 nm. This range is chosen because ophthalmic laser for therapeutic applications do not only emit light in the visible region but also in the near infrared region > 780 nm for example in laser photocoagulation with 810 nm [29], [30], [31], [32], [33], [34] or in near-infrared photoacoustic ophthalmoscopy with 1064 nm [35]. Therefore, we want to determine the reflectance properties of the eyewall in a wide spectral range from 350 to 1100 nm, since many wavelengths can be interesting for ophthalmological studies and we do not want to exclude any of them. In this work, the influence of the pigmentation of the eye on the reflectance spectra of 89 porcine eyes with different amount of pigmentation are investigated. To examine the effect of melanin on the reflectance spectrum, the absorbance spectrum of melanin is of interest and is measured in a spectral range between 350 and 1100 nm. Additionally, the absorption spectrum of porcine blood is recorded for the complete spectral range between 350 and 1100 nm, complementary to previous studies. With the absorption spectra of melanin and hemoglobin the reflection properties of the eyewall can be explained as well as the Sphere Multiplier. In our study, we present the Sphere Multiplier as a function of wavelength in a range of 350 - 1100 nm to determine the increase in photochemical and thermal retinal hazard. In addition to the wavelength, the pigmentation of the eye also plays a major role for the Sphere Multiplier, analogue to the reflection properties of the eyewall. Hence, the Sphere Multiplier of eyes with small and high amount of pigmentation is differentiated in this study and thus the increase in photochemical and thermal hazard for different pigmentation of the eye.

## Material and Methods

### Material

#### Porcine eyes

**Figure 1:**
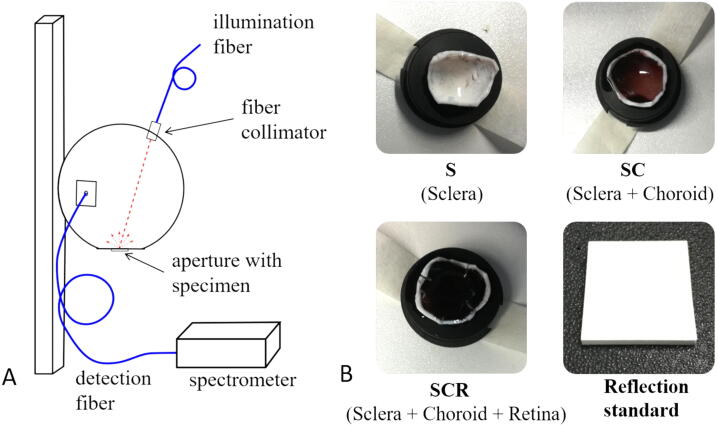
A: To measure the reflectance of a sample an integrating sphere is applied. An illumination fiber illuminates the sample, which is placed just below the aperture of the sphere, with an incidence angle of 20°. The reflected light is detected with a second fiber, which is connected to a spectrometer. B: Three different tissue layers are investigated, the sclera (S), the sclera combined with the choroid (SC) and all layers of the eyewall together, the sclera, choroid and retina (SCR). As reference, a reflecting standard is placed just below the aperture.

In this study, porcine eyes are used as tissue specimens. They are a good alternative to human eyes as they have many similarities in anatomy and physiology [36], [37], [38], [39], [40], [41]. For this, ex-vivo porcine eyes are obtained from a local slaughterhouse. They are stored in balanced salt solution (BSS of Alcon, Geneva (Switzerland)) and cooled at 8 °C. To avoid tissue deterioration measurements are performed within 6 h after enucleation [42]. Eyes, in which no more light transmission through the eyewall is visible, are sorted out because of suspected hemorrhage due to the removal procedure in the slaughterhouse. In total, 92 eyes are selected and divided into eyes with smaller amount of pigmentation (blue iris) and larger amount of pigmentation (brown iris). Reflectance measurements are performed on three different tissue types, sclera (S), sclera and choroid (SC) and sclera, choroid and retina (SCR) together. Therefore, a circular piece of the eyewall is cut out in the area of the equator of the eye. To prevent curling of the specimen, small cuts are made at the edge of the specimen (see Figure 1). For reflectance measurements of the sclera 38 eye are examined (15 and 23 eyes with blue and brown irises, respectively) and for measurements with sclera and choroid together, 36 eyes are investigated (11 and 25 eyes with blue and brown irises, respectively). For reflectance measurements of the whole eyewall (sclera, choroid and retina) 18 eyes are examined (9 eyes with blue irises and 9 eyes with brown irises).

#### Porcine hemoglobin

Fresh porcine blood is obtained from the same local slaughterhouse as the porcine eyes. According to Faber et al. the absorption spectrum of blood is mainly dominated by the hemoglobin absorption spectrum [43]. Therefore, to obtain a porcine hemoglobin like absorption spectrum, the absorbance of porcine blood is measured and the received spectrum is employed as relative hemoglobin absorption spectrum in the following analysis.

#### Melanin

Synthetic melanin is obtained from Sigma Aldrich in a powder form (Synthetic melanin, M8631-100MG of Sigma Aldrich) and stored at -20 °C.

### Experimental Set-Up

#### Reflectance of porcine eyewall

To measure the reflectance of the inner side of the eyewall an integrating sphere is used (Figure 1A). The integrating sphere (MSP REFLTRANS1 of Mountain Photonics GmbH, Landsberg am Lech (Germany)) is positioned with the 10 mm aperture facing down. The specimen to be examined are placed on a black circular block (Figure 1B) just below this aperture. To illuminate the sample, a bifurcated fiber (BFY400MS02 of Thorlabs, Newton (USA)) is applied to combine the light from a xenon lamp (LQX 1000 SC of Linos, Goettingen (Germany)) and a halogen lamp (SLS201 L/M of Thorlabs, Newton (USA)) to obtain a reflectance spectrum in a broad range from 350 to 1100 nm. To stabilize the output of the lamps they are warmed up of half an hour before the measurements start. The light enters the integrating sphere through a collimator and hits the sample with an incidence angle of 20°, interacts with the tissue and is partly reflected back into the inside of the integrating sphere. A second fiber (M114L01 of Thorlabs, Newton (USA)) detects the reflected light and transmits it to a spectrometer (SensLine AvaSpec 2048XL of Avantes, Apeldoorn (The Netherlands)). As a reference, a reflection standard manufactured of optical grade PTFE (MSP OPTFE BLOCK 50x50x5 mm^3^ of Mountain Photonics, Landsberg am Lech (Germany)) is applied and placed onto the aperture, which diffusely reflects the incident light.

To determine the amount of reflected light of the specimen three different spectra are necessary. The spectrum *P*, where the probe is placed just below the aperture, a reference spectrum *R*, where the reflection standard is placed just below the aperture, and a halo spectrum *H*. The halo spectrum is the spectrum of the scattered light that remains in the integrating sphere and could not exit through its aperture. During these three measurements, the light stays on. With these spectra, the reflectance ρ can be calculated as shown in equation (1).

(1) $$\rho =\frac{\text{P}-\text{H}}{\text{R}-\text{H}}$$

#### Absorbance of porcine hemoglobin and melanin

To measure the relative absorbance of porcine hemoglobin and melanin the samples are diluted in distilled water and put in a 1 ml UV-cuvette, respectively. As reference 1 ml pure distilled water is used. The absorbance is measured using a spectrometer (SPECORD PLUS of Analytik Jena, Jena (Germany)) in the wavelength range between 350 and 1100 nm.

### Integrating Sphere Model

The Sphere Multiplier *M* of an integrating sphere is given by Parretta and Calabrese (2013) [25] and represents the increase of radiance due to multiple reflections inside a sphere compared to a planar diffuser of the same size:

(2) $$\text{M}=\frac{1}{\left(1-\text{f}\right)\left(1-\rho \left(1-\text{f}\right)\right)}$$

The reflectance of the sample is ρ and *f* is the ratio of the open ports to the overall area of the sphere. In case of the eye *f* is the fraction of the size of the iris and pupil together divided by the surface of the eye: *f* = A_Iris_/A_Eye_ = 0.075, with different radii of 6 and 7.2 mm of the elliptic iris and with a diameter of the eye of 23.9 mm [38]. With *ρ* and *f* the Sphere Multiplier is calculated.

Due to these multiple reflections inside the eye, caused by its spherical shape, the irradiance inside the eye increases, compared to the irradiance that would be observed if there wouldn’t be this spherical shape. In ophthalmologic surgery the illumination of the ocular fundus is performed with either hand-held endoillumination fibers or chandeliers [44], [45], [46], [47]. In this study, the irradiances of different typically used illumination fibers in ophthalmology are measured with an integrating sphere according to the standard ISO 15752:2010 [48]. These irradiances are multiplied with the photochemical and thermal hazard weighting functions, A(λ) and R(λ), to determine the photochemical weighted irradiance E_A-R_ and the thermal weighted irradiance E_VIR-R_ on the retina (equation 3 and 4) [6]. The starting and final value of the sum differ from [6] because the illumination fibers only emits light between 380 and 780 nm.

(3) $${\text{E}}_{\text{A}-\text{R}}=\overset{700\text{nm}}{\underset{380\text{nm}}{\sum }}{\text{E}}_{\lambda }\times \text{A}\left(\lambda \right)\times \Delta \lambda$$

(4) $${\text{E}}_{\text{VIR}-\text{R}}=\overset{780\text{nm}}{\underset{380\text{nm}}{\sum }}{\text{E}}_{\lambda }\times \text{R}\left(\lambda \right)\times \Delta \lambda$$

To measure the emission of these different illumination fibers they are combined with either a halogen lamp (Accurus Surgical System version 600 DS from Alcon Laboratories Inc. (Fort Worth, TX, USA)) or a xenon light source (Xenon BrightStar from D.O.R.C. (Zuidland, The Netherlands)) depending on the illumination fiber. Some fibers can only be connected to the halogen lamp and some can only be used in combination with the xenon lamp, depending on which type of adapter they have. These two light sources are typically used in ophthalmic surgery and emit light in the region between 380 and 780 nm. The emitted irradiances of these fibers were measured with another integrating sphere (ISP 250 UV, Instrument Systems Optische Messtechnik GmbH, Munich (Germany)) and an array spectrometer (CAS 140D, Instrument Systems Optische Messtechnik GmbH, Munich (Germany)) in the range between 380 and 780 nm. The intensities of the light sources are set to the maximum possible intensity in combination with the used fibers.

The irradiances E*_A-R_ and E*_VIR-R_, due to multiple reflections, are calculated by weighting equation (3) and (4) with M(λ), given in equation (5) and (6), where M(λ) is the Sphere Multiplier depending on the wavelength and E_λ_ is the irradiance of the fiber without reflections.

(5) $$\text{E}{*}_{\text{A}-\text{R}}=\overset{700\text{nm}}{\underset{380\text{nm}}{\sum }}{\text{E}}_{\lambda }\times \text{A}\left(\lambda \right)\times \text{M}\left(\lambda \right)\times \Delta \lambda$$

(6) $$\text{E}{*}_{\text{VIR}-\text{R}}=\overset{780\text{nm}}{\underset{380\text{nm}}{\sum }}{\text{E}}_{\lambda }\times \text{R}\left(\lambda \right)\times \text{M}\left(\lambda \right)\times \Delta \lambda$$

The increase of the photochemical and thermal hazard for the retina is given by ΔE_A-R_ and ΔE_VIR-R_, respectively, and is calculated according to equation (7) and (8).

(7) $$\Delta {\text{E}}_{\text{A}-\text{R}}=\frac{{\text{E}}_{\text{A}-\text{R}}}{\text{E}{*}_{\text{A}-\text{R}}}$$

(8) $$\Delta {\text{E}}_{\text{VIR}-\text{R}}=\frac{{\text{E}}_{\text{VIR}-\text{R}}}{\text{E}{*}_{\text{VIR}-\text{R}}}$$

To illustrate the resulting reduction in operation time, the exposure time is calculated for the illumination fiber (Combined 23 gauge Eckardt Multi-Fiber Endoillumination from D.O.R.C. (Zuidland, The Netherlands)) in combination with the xenon light source (Xenon BrightStar from D.O.R.C. (Zuidland, The Netherlands)).

### Statistical analysis

To perform a statistical analysis SPSS Statistics (SPSS Statistics Version 25 of IBM, Armonk (USA)) is used. To determine the difference in reflection between eyes with blue and brown irises the area under the curve (AUC) between 350 and 1100 nm and between 380 and 500 nm is calculated for each sample The AUC between 350 and 1100 nm is calculated to get an overall impression of the reflectance properties of the tissue, whereas the AUC between 380 and 500 nm is calculated due to the fact that this range is the most important one for the calculation of the photochemical retinal hazard. For these AUCs a Mann Whitney-U-Test is performed. P < 0.05 is considered as statistically significant.

## Results

### Reflectance of the inner eyewall in the range between 350 and 1100 nm

**Figure 2:**
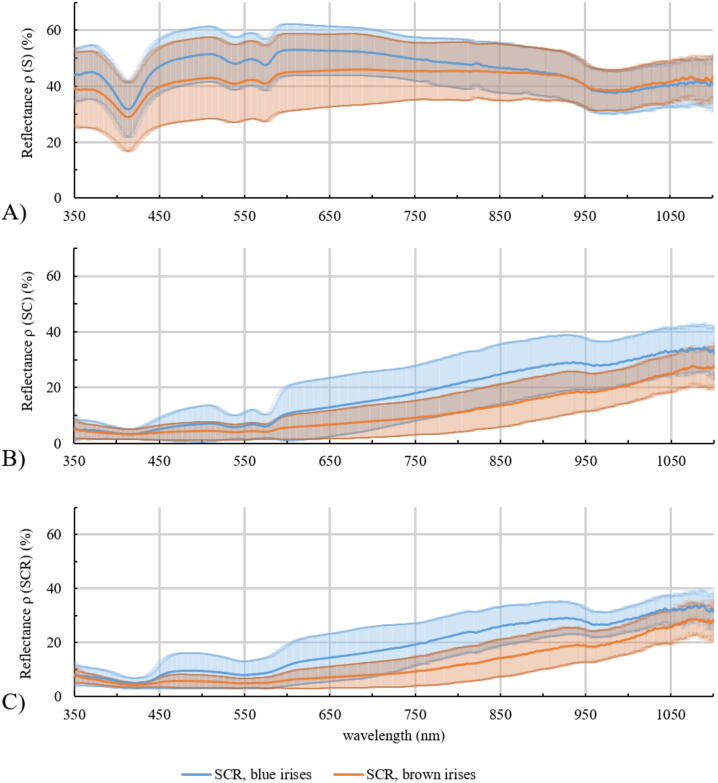
Wavelength-dependent reflectance in the range of 350 to 1100 nm of A: Sclera, B: Sclera and choroid and C: Sclera, choroid and retina together. The blue curve represents the data of less pigmented eyes, the brown curve represents data of highly pigmented eyes, with corresponding standard deviations.

**Figure 3:**
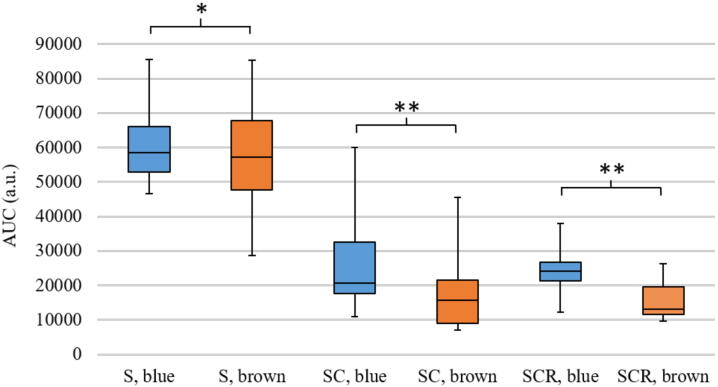
The calculated area under the curve (AUC) of the reflectance spectra between 350 and 1100 nm are shown as boxplots for eyes with blue irises in blue and for eyes with brown irises in brown, for different tissue layers S (sclera), SC (sclera + choroid) and SCR (sclera + choroid + retina). The medians of the blue boxes are significantly higher than that of the brown boxes (t-test: *≙ p < 0.05 and **≙ p < 0.001).

**Figure 4:**
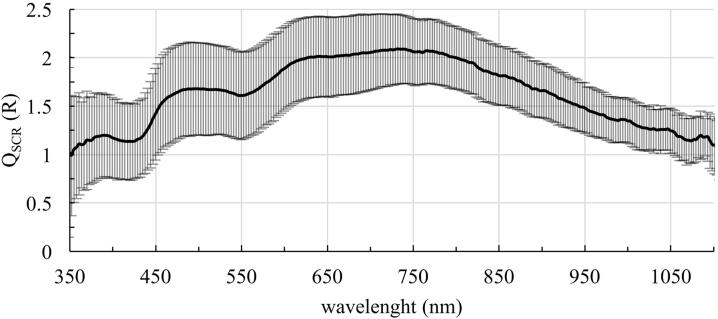
Quotient Q_SCR_(R) of the reflectance of the inner eyewall (sclera, choroid and retina) of eyes with blue and brown irises with standard deviation in the range of 350 – 1100 nm.

The reflectance is calculated for the sclera, S (Figure 2A), sclera and choroid, SC (Figure 2B) and sclera, choroid and retina, SCR (Figure 2C). The samples are divided in eyes with blue irises (blue curve) and eyes with brown irises (brown curve) with corresponding standard deviations. The sclera is the most reflective layer of the eyewall. It reflects on average between 31.68 and 53.05% of the incoming light in the range of 350 and 1100 nm for eyes with blue irises and between 28.92 and 46.05% for eyes with brown irises. With additional choroid the mean reflectance in the visible spectral range decreases very strongly and also in the near infrared range (NIR) the reflectance is smaller than without the choroid. The mean reflectance ranges between 3.23 and 35.80% for eyes with blue irises and 3.16 and 28.96% for eyes with brown irises, in the shown spectral range. Considering all parts of the eyewall the mean reflectance varies between 4.85 and 34.77% and between 4.24 and 30.37% for eyes with blue and brown eyes, respectively. The minimum and maximum reflectance for different tissues is listed in Table 1. Considering the influence of the pigmentation of the eye on its reflectance, it is demonstrated that highly pigmented eyes reflect less light than less pigmented eyes. For each reflection spectrum the area under the curve (AUC) is calculated between 350 and 1100 nm. The mean AUCs for each tissue is given in Table 1. In Figure 3 the calculated AUC for the respective tissue layer and eye color is depicted as a boxplot with corresponding median. It can be observed that the AUCs for eyes with blue irises are higher than for eyes with brown irises, which indicates a higher reflectance. These AUCs are statistically evaluated with SPSS Statistics. Performing a Mann-Whitney-U-Test between these values for blue and brown eyes shows that blue eyes have a higher AUC than brown eyes. Therefore, the pigmentation of the eye has a statistically significant effect on its reflectance properties. Additionally, the AUC between 350 and 500 nm is calculated and a Mann-Whitney-U-Test is performed. The corresponding p-values (for the AUC between 350-1100 nm and between 380 and 500 nm) are also given in Table 1. The AUCs of the reflexion spectrums between 350 and 1100 nm are statistically different between blue and brown eyes for all investigated layers, whereas the AUCs between 350 and 500 nm just show a significant difference between blue and brown eyes for the sclera (S). The AUCs of the reflection spectrums of sclera and choroidea (SC) and the whole eyewall (SCR) are not significantly different for blue eyes compared to brown eyes. The increase in reflection for blue eyes compared to brown eyes is shown in Figure 4. The ratio of the reflectance R of blue and brown eyes, Q_SCR_(R) = R_blue_/R_brown_, is given for wavelengths between 350 and 1100 nm. In Figure 4 it can be seen that blue eyes exhibit an increase in reflection by a factor of between 1.02 and 2.10.

**Table 1:** The minimum reflectance, the maximum reflectance and the calculated area under the curve (AUC) with standard deviation for different layers of the eyewall (sclera (S), sclera + choroid (SC) and sclera + choroid + retina (SCR)) are compared between eyes with blue and brown irises with resulting p-value of the Mann-Whitney-U-Test in the wavelength range between 350 and 1100 nm and 380 and 500 nm. If p< 0.05 the AUCs between blue and brown eyes are statistically significant different.

	Minimum reflectance [%]		Maximum reflectance [%]		AUC (350-1100 nm) [× 10^3^]		p-value (350-1100 nm)	p-value (380-500 nm)
	Blue	Brown	Blue	Brown	Blue	Brown		
S	31.68 ± 10.05	28.92 ± 12.29	53.05 ± 9.20	46.05 ± 12.61	60.30 ± 9.31	55.46 ± 13.15	0.0331	0.011
SC	3.48 ± 2.11	3.59 ± 2.38	36.60 ± 8.05	30.05 ± 7.78	25.23 ± 11.83	16.68 ± 11.05	0.0005	0,095
SCR	4.85 ± 1.93	4.24 ± 0.93	34.77 ± 5.97	30.37 ± 6.95	24.63 ± 7.42	15.84 ± 5.49	0.0003	0.062

### Absorbance of melanin and hemoglobin in the range between 350 and 1100 nm

**Figure 5:**
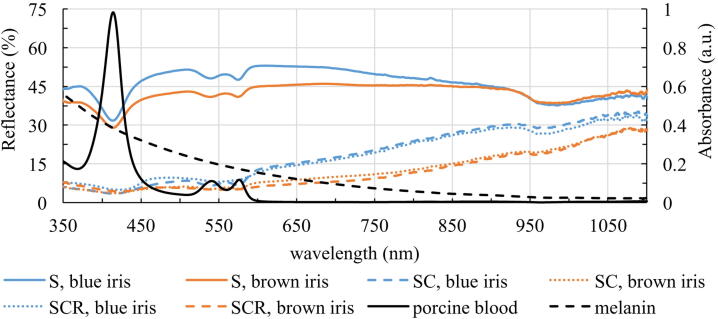
Reflectance of S (sclera, solid line), SC (sclera and choroid, dashed line) and SCR (sclera, choroid and retina, dotted line) for eyes with blue and brown irises analogue to Figure 2. Additionally, the absorbance spectra of porcine hemoglobin (black solid line) and melanin (black dashed line) are given.

To determine the influence of hemoglobin and melanin to the reflectance of the inner eyewall the absorbance spectra of porcine hemoglobin and synthetic melanin are recorded. The relative absorbance of hemoglobin (black solid line) and melanin (black dashed line) in the range from 350 to 1100 nm are plotted in Figure 5. The melanin absorbance decreases with increasing wavelength. The absorbance of porcine hemoglobin also decreases with increasing wavelength with three exceptions. At 414 nm there is a large absorbance peak as well as there are two smaller peaks at 541 and 576 nm. Additionally, the reflectance spectra of the different tissues of the eyewall are illustrated to compare the absorbance spectra with the reflectance. The reflectance of the sclera (solid line), sclera and choroid (dashed line) and sclera, choroid and retina together (dotted line) are shown for eyes with blue irises (blue curve) and eyes with brown irises (brown curve), respectively. It can clearly be seen that the reflectance of the tissue has a local minimum where the absorbance spectra of hemoglobin has its local maximum. It also can be observed that the reflectance of SC and SCR decreases with increasing melanin absorbance.

### Integrating sphere model in the range between 350 and 1100 nm

**Figure 6:**
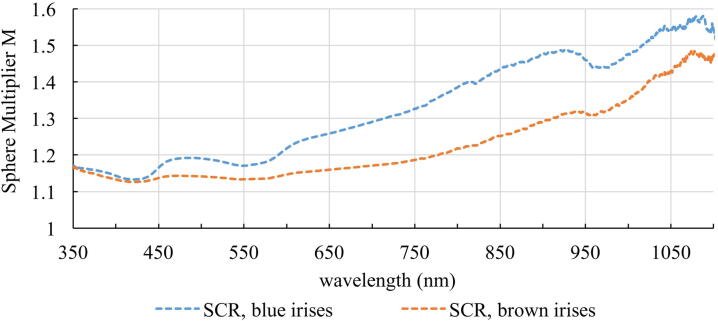
Calculated Sphere Multiplier M for different tissues SCR depending on the wavelength and on the pigmentation of the eye.

For determining the Sphere Multiplier *M* for eyes with blue and brown irises equation (2) is calculated by using the reflectance values from Figure 2 and *f* = 0.075. The results are shown in Figure 6. For the complete eyewall (SCR) the Sphere Multiplier increases with increasing wavelength. Due to this behaviour, the increase in photochemical and thermal retinal hazard will be different, as the photochemical hazard is mainly caused by short-wavelength light and the thermal hazard by longer wavelengths.

**Figure 7:**
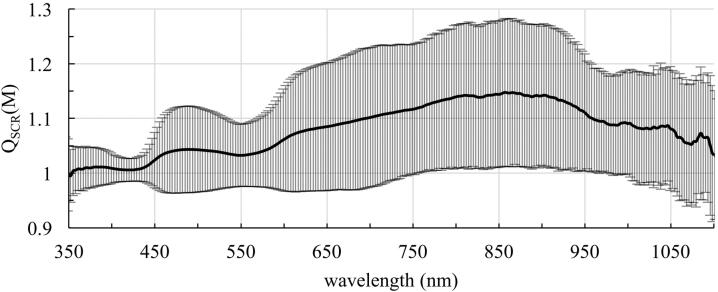
Quotient Q_SCR_ (M) of the reflectance of the inner eyewall (sclera, choroid and retina) of eyes with blue and brown irises with standard deviation in the range of 350 – 1100 nm.

It is observed that the sphere Multiplier not only depends on the wavelength but also on the pigmentation of the eye. The Sphere Multiplier is larger for eyes with blue irises compared to brown irises. The increase of the Sphere Multiplier for blue eyes compared to brown eyes is shown in Figure 7. The ratio of the Sphere Multiplier *M* of blue and brown eyes, Q_SCR_ (M) = M_blue_/M_brown_, is given for wavelengths between 350 and 1100 nm. The Sphere Multiplier, and therefore the increase in irradiance, in blue eyes is higher than in brown eyes by a factor between 1.003 and 1.151.

### Increase of retinal hazard in the range between 380 and 780 nm

The measured irradiances of various typical used illumination fibers in ophthalmology according to the standard ISO 15752:2010 [48] indicate that the specified limit value for the photochemical hazard to the retina is exceeded for all fibers, except one. If the limit value is exceeded, the exposure time must be calculated according to the standard. The operator must perform the operation within this time and must not exceed it.

**Figure 8:**
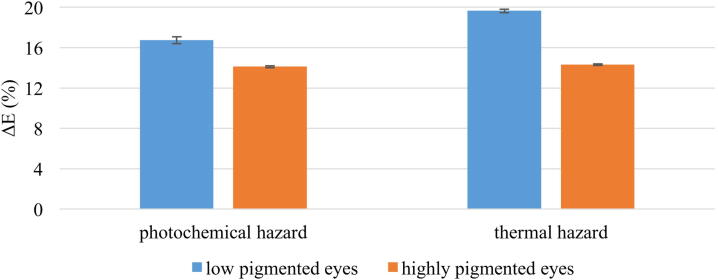
The mean value of the intraocular increase ΔE considering potential photochemical and thermal hazard for the retina due to multiple reflections inside the eye is given with standard deviation. The results for low pigmented eyes (blue bars) and for highly pigmented eyes (brown bars) are illustrated.

With this Sphere Multiplier the increase in potential retinal hazard can be calculated according to equation (5) and (6) for the photochemical and thermal hazard. For this calculation the Sphere Multiplier is only important in a range between 380 and 780 nm, as the illumination light source only emit in this wavelength range. Due to the spherical anatomy of the eye the photochemical retinal hazard of highly pigmented eyes increases by (14.11 ± 0.09)% and of low pigmented eyes by (16.75 ± 0.35)%, thus reducing the exposure time by these factors. As example, for measurements with the Endoillumination fiber(Combined 23 gauge Eckardt Multi-Fiber Endoillumination) and the xenon lamp, both from D.O.R.C., the exposure time decreases from around 40 min to 34 min for less pigmented eyes and 35 min for highly pigmented eyes for a distance of 5 mm between the fiber tip and the retina. The thermal retinal hazard increases by (14.30 ± 0.07)% for highly pigmented eyes and by (19.65 ± 0.17)% for lightly pigmented eyes. Figure 8 gives a good overview of the increase in photochemical and thermal hazard in low pigmented eyes compared to highly pigmented eyes.

## Discussion

The reflectance measurements of the inner side of the porcine eyewall resulted in a significant higher reflectance for less pigmented eyes compared to strong pigmented eyes between 350 and 1100 nm. Here, the reflectance of the sclera is the largest. The sclera also contains some blood [20], which can be explained by the hemoglobin peaks at 414, 541 and 576 nm in the reflectance spectrum (Figure 2A). The peak at 975 nm is due to the high absorption peak of water [49] present in the sclera.

Considering all layers of the eyewall, the reflectance becomes smaller, due to hemoglobin and melanin absorption, which are located in the choroid and retina. The reflectance of the inner side of the eyewall is between 4.85 and 34.77% for eyes with blue irises and between 4.24 and 30.37% for eyes with brown irises. Additionally, the reflectance increases with increasing wavelength. Considering the pigmentation of the eye our data reveals significant higher reflectance values for eyes with blue irises compared to eyes with brown irises in the range between 350 and 1100 nm.

The differences in the reflection spectra for different pigmented eyes are partly due to the melanin content in the eye. Melanin absorbs more light in the short-wavelength range compared to longer wavelengths (Figure 5). Therefore, the reflectance spectrum of the ocular fundus is the smallest in the small-wavelength region and increases towards larger wavelengths. This behavior can also explain the difference in reflectance measurements between less and more pigmented eyes. Highly pigmented eyes contain more melanin in the choroidal layer of the eye which absorbs more light and therefore reflects less. As a consequence, the light can be reflected more often in blue eyes than in brown eyes leading to an increase in irradiance on the retina. Due to reflections in the sclera, the choroid and the RPE layer, the retina is again irradiated by the back-reflected light. In blue eyes, the retina is thus transilluminated with greater portion of light than in brown eyes, where a greater portion is absorbed. Measurements of Kadam et al. (2011) revealed that for the melanin content in the choroid-RPE it is rat > porcine ∼ bovine > pigmented rabbit > albino rabbit [15], which indicates that pigmented eyes have a greater melanin content in the choroid-RPE than non-pigmented eyes. These data are expanded by Menon et al. (1992). They prove that the choroid-RPE melanin content for human is between that of pigmented rabbit and albino rabbit of the measurements from Kadam et al. (2011) and is therefore smaller compared to choroidal-RPE in porcine eyes [18]. This result is also supported by Durairaj et al. (2012), who also claim that the choroidal-RPE melanin content is higher in porcine eyes compared to human eyes [50]. These results indicate an even higher reflectance of the human ocular fundus as described in this work, because less light is absorbed due to lower melanin content. This leads to an even higher increase in retinal hazard for human eyes.

Another influence of the increase in reflection of the ocular fundus for larger wavelengths is the strong decrease in hemoglobin absorption in this range. In our study we performed an absorbance measurement of porcine blood, which is mainly dominated by hemoglobin absorption, and covered the complete spectral range between 350 and 1100 nm, which extends the spectral range of porcine hemoglobin absorption in the literature [22], [23].

Due to the reflection properties of the ocular fundus and its spherical shape the intraocular irradiance increases compared to if the eye would be unfolded. With the spherical geometry of the eye light can often hit the same part of the retina and therefore increase its hazard. The Sphere Multiplier of the ocular fundus reaches values between 1.13 and 1.58 for a wavelength range between 350 – 1100 nm. Our study expands the results of Koelbl et al. (2019) [24] and reveals the wavelength-dependence of the Sphere Multiplier. It is observed that the Sphere Multiplier increases with increasing wavelength due to the absorption properties of melanin and hemoglobin. To our knowledge, this study is the first to show the increase in irradiation inside porcine eyes as a function of wavelength. With this wavelength-dependence a special focus can be placed on the increase in photochemical and thermal hazard to the retina. Due to the anatomically shape of the eyeball the photochemical hazard increases about 14% (brown eyes) and 17% (blue eyes), and the thermal hazard increases about 14% (brown eyes) and 20% (blue eyes), due to multiple reflections inside the eye. Therefore, the maximum exposure time decreases. Since the measurements of irradiances with typical ophthalmic optical fibers show that the limits in the standard DIN EN ISO 15004-2:2007 [6] are exceeded and the surgeon is thus limited in the time of application, he will be even more limited with this increase in irradiance. Measurements for validation of the illumination fibers are performed in reference set-ups where no reflections can take place. Therefore, the effective irradiance in the eye is higher than assumed in the reference measurement of the standard due to multiple reflections and the maximum exposure time is actually reduced. The exemplarily measurement with the xenon lamp and the illumination fiber “Combined 23 gauge Eckardt Multi-Fiber Endoillumination”, with a distance of 5 mm between the fiber tip and the retina, demonstrated that the maximum exposure time is 40 min without considering the reflection characteristic of the eye and decreases to 34 min when considering these properties for blue eyes. Vitreoretinal surgery is a time-consuming procedure and may last between 30 minutes and a few hours. In 2017, Alitair da Silva Costa evaluated the duration time of operative time of different surgical specialties. An ophthalmological operation takes about 111 ± 86 min on average [51]. Therefore, 34 min are insufficient to perform a vitrectomy, or even 40 min are generally not enough. Since the surgeon can also get closer to the retina at times with the illumination fiber, the effective time allowed is even shorter. However, most clinicians in retinal surgery dim the light as much as possible to cause as little damage to the retina as possible. In our study, we chose the maximum adjustable intensity to cover the worst-case during surgery. Since the light sources are adjustable in brightness, in reality not the maximum brightness of the lamp would be used, but a lower intensity, with which the surgeon can still distinguish between fine structures inside the eye, but the hazard to the retina is reduced. We also chose the highest intensity to have the advantage of higher measurements accuracy with smaller influence of spectrometer noise, which is an issue at the limits of the detection range. As the intensity increases, the signal at the spectrometer increases, including noise signal. However, the signal-to-noise ratio also increases, resulting in a more accurate measurement.

Another method to reduce the hazard to the retina could be the use of light sources with a non-continuous spectrum. By reducing the portion of wavelengths in the spectrum that are not necessary for imaging in the eye, the radiation in the eye is reduced. Especially the reduction of the short-wavelength part of the spectrum can lead to a reduction of the photochemical ability. For example, white LEDs with relatively narrow blue emission peaks are less dangerous than xenon light sources which consists of the same blue emission peak and an additional portion of shorter and longer wavelength light [27], [52]. The thermal hazard can also be decreased by a non-continuous spectrum, since all visible light can contribute to thermal damage. However, these effects are strongly dependent on the level of irradiance. If the irradiance is too high even for non-continuous light sources, they can be more dangerous to the retina than light sources with a continuous spectrum.

When performing ophthalmic procedures with light sources, which emit light in the wavelength range around 960-980 nm, the absorption properties of the vitreous body should be included in the hazard evaluation, as it has a high absorption in this wavelength range, which is due to its high water content. Based on to these absorption properties, light in this spectral range would be attenuated and thus contribute less to the retinal hazard than without considering the vitreous body. In our evaluation, the vitreous body was not considered, since the light sources used in our study only emit light in the wavelength range between 380 and 780 nm. Another reason, for not considering the absorption properties of the vitreous body, is that some surgical procedures are performed after a vitrectomy, when no vitreous body is present in the eye.

In this study, the reflectance properties of the porcine eyewall are determined in the equatorial region of the porcine eye. The reflectance properties in the region of the optic nerve, the fovea or the pars plana may differ from the results presented in this study. This is because the thickness of the eyewall and the pigment concentration vary in different parts of the eye. Two additional factors influencing the reflection properties of the eyewall could be the angle of incidence and the polarization of the light. The latter plays only a minor role, since most ophthalmological light sources emit non-polarized light. In case of laser applications in the eye, the reflection properties of the eyewall depend on the polarization angle, following the Fresnel equations. However, the effect of polarization on the reflection of the eyewall and the incident angle of the light on the retina were not investigated in this study. In our measurements the light fell on the retina at approximately the same angle as it usually does during eye surgery. To measure the angular dependence of the reflection in the eye was not possible with our set-up, but is a task which could be investigated in the future.

Our study proves that the pigmentation of the eye has an influence on the Sphere Multiplier M. In less pigmented eyes the Sphere Multiplier of the eyeball is higher as for highly pigmented eyes. However, this effect is very strongly dependent on the wavelength. This is illustrated clearly in Figure 7. The surgeon should be aware that the pigmentation of the eye should have an influence on the duration of the operation. In previous studies, for example, it was shown that the risk of developing senile macular degeneration [53], [54], [55], as well as an increased risk for uveal melanoma [56], [57], [58], is higher for people with light irises than for darkly pigmented people. Therefore, when illuminating the eye, it is important to consider its pigmentation so that the limits are not exceeded. As a consequence for posterior segment surgery, eye color could be a parameter in the settings for illumination intensity. If the settings “weakly pigmented eye” and “strongly pigmented eye” were available, the intensity of the illumination intensity or the maximal exposition time should be adapted. In the setting “weakly pigmented eye” the light intensity should be lower as for the setting “strongly pigmented eye”.

In the literature, unexplained vision loss after eye surgery is often reported. Despite a good understanding of eye physiology and diagnostic capabilities, there are still cases of unexplained vision loss [59], [60], [61], [62], [63], [64]. The results of this study may be a new explanation for unexpected visual acuity failures.

## Conclusion

Our study reveals that the reflection properties of porcine eyes depend strongly on the wavelength and on the pigmentation of the eye. The wavelength- and pigmentation-dependency can be explained by the absorption spectrum of hemoglobin and melanin located in the eyewall. The reflection of the eyewall has a great influence on the Sphere Multiplier, which represents the increase of irradiance due to multiple reflections inside a sphere compared to a plane of the same size and reflection properties. Due to these multiple reflections and their wavelength-dependency, the photochemical and thermal hazard for the retina increases in comparison to if no reflections would be considered. The anatomy and pigmentation of the eye plays an important role for the intraocular irradiance on the retina and for the maximum exposition time during vitreoretinal surgery. These results indicate that the limit values specified in the standard DIN EN ISO 15004-2:2007 [6] are estimated to be too weak and the exposition time too long, as the irradiance in the eye is higher than assumed.

## Acknowledgment

### Funding

This work was financially supported by the German Federal Ministry of Economics and Technology within the ZIM joint project “Safe Light” (grant number ZF4137902AK9). This article does not contain any studies with human participants or animals performed by any of the authors.

### Conflict of Interest

The authors declare that they have no known competing financial interests or personal relationships that could have appeared to influence the work reported in this paper.
